# The Retromer Subunit CfVps29 Is Involved in the Growth, Development, and Pathogenicity of *Colletotrichum fructicola*

**DOI:** 10.3390/jof8080835

**Published:** 2022-08-10

**Authors:** Sizheng Li, Xiya Li, He Li

**Affiliations:** 1 Key Laboratory of National Forestry, Grassland Administration on Control of Artificial Forest Diseases and Pests in South China, Central South University of Forestry and Technology, Changsha 410004, China; 2 Hunan Provincial Key Laboratory for Control of Forest Diseases and Pests, Central South University of Forestry and Technology, Changsha 410004, China

**Keywords:** *Camellia oleifera*, *Colletotrichum fructicola*, CfVps29, pathogenicity

## Abstract

*Camellia oleifera* is an edible oil tree species native to China. Anthracnose is a common disease of *Ca. oleifera*, which reduces the production of the trees and brings huge economic losses. We have previously identified the fungus *Colletotrichum fructicola* as the major pathogen of anthracnose in *Ca. oleifera*. The retromer complex participates in the intracellular retrograde transport of the cargos from the endosome to the trans-Golgi network in the eukaryotes. Vacuolar protein sorting 29 is a subunit of the retromer complex. Targeted *CfVPS29* gene deletion revealed that CfVps29 is involved in growth, conidiation, and the response to cell wall stress. We further found that the Δ*Cfvps29* mutant was minimally pathogenic to *Ca. oleifera* leaves, as a result of its defect in appressorium formation. This study illustrated the crucial functions of CfVps29 in the development, cell wall stress response, and pathogenicity of *C. fructicola* and, therefore, identified it as a potential fungicide target for the control of anthracnose.

## 1. Introduction

*Camellia oleifera* (tea-oil tree) is native to China and is a unique woody plant that has been cultivated in many areas of southern China for over two thousand years, mainly for its high-quality cooking oil. Anthracnose is a major disease of *Ca. oleifera*, caused by the *Colletotrichum* species [1]. The buds and fruits of diseased plants always drop as a result of this disease, resulting in huge economic losses. *Colletotrichum fructicola* is the main epidemic pathogen causing anthracnose in *Ca. oleifera* [1].

The retromer complex consists of multiple vacuolar protein sorting proteins, which are associated with the cytoplasmic surface of the endosomes and mediate the retrograde transport of the transmembrane cargo in endosome-to-Golgi transport [2,3]. Vps29 is a key component of the cargo-binding core complex of the retromer, a protein assembly with diverse roles in the transport of receptors within the endosomal system. Vps29 has a fold related to the metal-binding phosphatases and mediates interactions between the retromer and other regulatory proteins. In yeast, Vps29p is essential for the association of the Vps29p-Vps35p-Vps26p core complex with the sorting nexins [4]. In plant pathogenic fungi, Vps29 regulates pathogenicity in *Magnaporthe oryzae* and *Fusarium graminearum* [4,5].

As a component of the retromer complex, the role of Vps29 in *C. fructicola* is unclear. In this study, CfVps29 was identified in *C*. *fructicola*, and the knockout mutants of the protein-coding gene *CfVP29* were obtained through a gene replacement approach. Further analysis of the biological phenotype revealed the function of CfVps29 in *C*. *fructicola* and provided an investigative basis for the development of new fungicides targeting this protein.

## 2. Materials and Methods

### 2.1. Strains and Culture Conditions

The CFLH16 strain was used as the WT of *C. fructicola*. The WT strain, the gene deletion mutants ∆*Cfvps29*-2 and ∆*Cfvps29*-12, and the complemented strain Δ*Cfvps29*-2C were maintained on complete medium (CM) in darkness at 28 °C for mycelial growth. The *Escherichia coli* competent cells were purchased from TsingKe Biological Technology Co. The XK-125 yeast strain and pYF11 plasmid used in complementation were provided by Professor Zhang (Nanjing Agricultural University, China).

### 2.2. Phylogenetic Analysis and Domain Prediction

The sequences of the Vps29 proteins of *C. truncatum*, *Fusarium austroafricanum*, *C. karsti*, *M. oryzae*, *Saccharomyces cerevisiae*, and *C. fructicola* were obtained from the NCBI (https://www.ncbi.nlm.nih.gov/, accessed on 18 January 2022). The phylogenetic tree was constructed using Mega 7.0. The protein sequences were submitted to MEME (https://meme-suite.org/, accessed on 22 January 2022) for motif prediction. To predict the conserved domain of the proteins, a CD-search from NCBI was carried out, and the analysis results were displayed using TBtools software (Guangzhou, China).

### 2.3. Targeted Gene Deletion and Complementation

*CfVPS29* gene deletion was used as the one-step replacement strategy, as in our previous description [6]. First, two ~1.0 kb sequences flanking the targeted gene were amplified and overlapped with the flanks of the hygromycin resistance cassette, respectively (Figure S2A). Then, the resulting ~3.4 kb fragments were introduced into protoplasts of the WT. For the complementation strain, the CfVPS29-9F and CfVPS29-10R primers were designed, and the complementary fragments that included the CfVPS29 gene and promoter sequences were amplified by PCR. The PCR products were purified and then cotransformed into the yeast competent cell XK-125 with the pYF11 vector that was linearized using XhoI (containing the bleomycin(BLE)-resistant gene and green fluorescence protein(GFP) gene) to form the complementary carrier of pYF11: *CfVPS29*. The yeast cells were cultured on SD-Trp medium for screening, and the primers CfVPS29-7F/GFP-R were used for the PCR identification of positive clones. The successfully fused plasmids were then transformed into the *Escherichia coli* JM109 competent cells. PCR was used for the identification and sequencing of the *E. coli*-positive clones. The complementary carriers with the correct sequences were transformed into the protoplasts of the mutant. The transformants that could grow on a BLE-containing culture medium were screened by fluorescent microscopy and further confirmed by PCR (Figure S2B). The primers used in this article are listed in Table 1.

### 2.4. Growth, Conidiation, and Appressoria Formation

The WT, ∆*Cfvps29*-2, ∆*Cfvps29*-12, and complemented strain Δ*Cfvps29*-2C were inoculated on CM and potato dextrose agar(PDA) plates at 28 °C in the darkness for 3 days, and the colony diameters were measured and statistically analyzed. For the conidiation assays, the strains were cultured in 100 mL of liquid-shaking potato dextrose broth (PDB) for 3 days, then the conidia were collected and quantified using a microscope. For the appressorial assays, the conidia were collected and adjusted to 10^5^ spores/mL. The conidial suspensions were placed on hydrophobic coverslips and incubated under humid conditions at 28 °C for appressorium formation. The appressoria were treated with 20 μL of glycerin (2 M), and the collapse rates were estimated.

### 2.5. Pathogenicity Assays

The conidial suspensions (1 × 10^6^ spores/mL) of the strains were inoculated onto the leaves of *Ca. oleifera*. The inoculated leaves were kept under high-moisture conditions at 28 °C. After incubation for 3–5 days, the lesions were observed and measured.

### 2.6. Cell Wall Integrity Assays

The strains were inoculated onto CM with 0.1% SDS and cultured at 28 °C. The colony diameters were measured, and the inhibition rates were statistically analyzed. The young mycelia were stained with 10 mg/mL Calcofluor white (CFW) for 10 min without light, washed with distilled water, and observed and photographed under the fluorescence microscope (Zeiss Axio Observer 3, Jena, Germany).

### 2.7. Real-Time Quantitative Reverse Transcription PCR Analysis

Total RNA was extracted using the RNAsimple Total RNA Kit (TIANGEN, Beijing, China), including DNase treatment. And cDNA synthesis was carried out using the HiScript^®^ 1st Strand cDNA Synthesis Kit (Vazyme, Nanjing, China). Real-time quantitative reverse transcription PCR (RT-qPCR) was performed to detect the expression level of the chitin synthase genes, CHSs, with the primer pairs (Table 1), and Actin F/R was used for amplification of the actin gene. SYBR green-based quantitative PCR was performed using a Quant Studio 3 machine (Applied Biosystems, Waltham, MA, USA). The comparative CT method was used for the quantitative comparison.

### 2.8. Statistical Analysis

All experiments were carried out at least three times, and each treatment had three replicates. All data were expressed as mean ± standard deviation (SD) and analyzed using SPSS 26.0 (Shenzhen, China). An analysis of variance (ANOVA) was carried out, followed by Duncan’s new multiple range test, *p* < 0.01 or *p* < 0.05.

## 3. Results

### 3.1. Identification and Phylogenetic Analysis of CfVps29

We identified a protein homologous to Vps29 of *S. cerevisiae* and named it CfVps29. The full length of the *CfVPS29* gene was 727 bp, which encoded 200 amino acids. We also collected CfVps29 homolog sequences in other fungi using BLASTP analysis. The phylogenetic analysis showed that the CfVps29 had higher homology with that of *C. truncatum* (Figure S1A). The domain prediction showed that CfVps29 shared five conserved motifs with other filamentous fungi, all of which possessed the MPP_Vps29 domain, and three conserved motifs with *S. cerevisiae*, while *S. cerevisiae* had the MPP superfamily domain (Figure S1B–D).

### 3.2. Generation of VPS29 Gene Deletion and Complemented Strains

The primers *CfVPS29*-5F and H855R were used and the amplification of a single band was obtained, while the use of the primers *CfVPS29*-7F/8R did not show any amplification, indicating that the *CfVPS29* gene was deleted in the mutants of Δ*Cfvps29*-2 and Δ*Cfvps29*-12 (Figure S2B). The bleomycin-resistant transformants were selected and confirmed using fluorescence and PCR. Then, the complemented strain Δ*Cfvps29*-2C was obtained (Figure S2B).

### 3.3. CfVPS29 Is Required for Development and Pathogenicity

**Figure 1:**
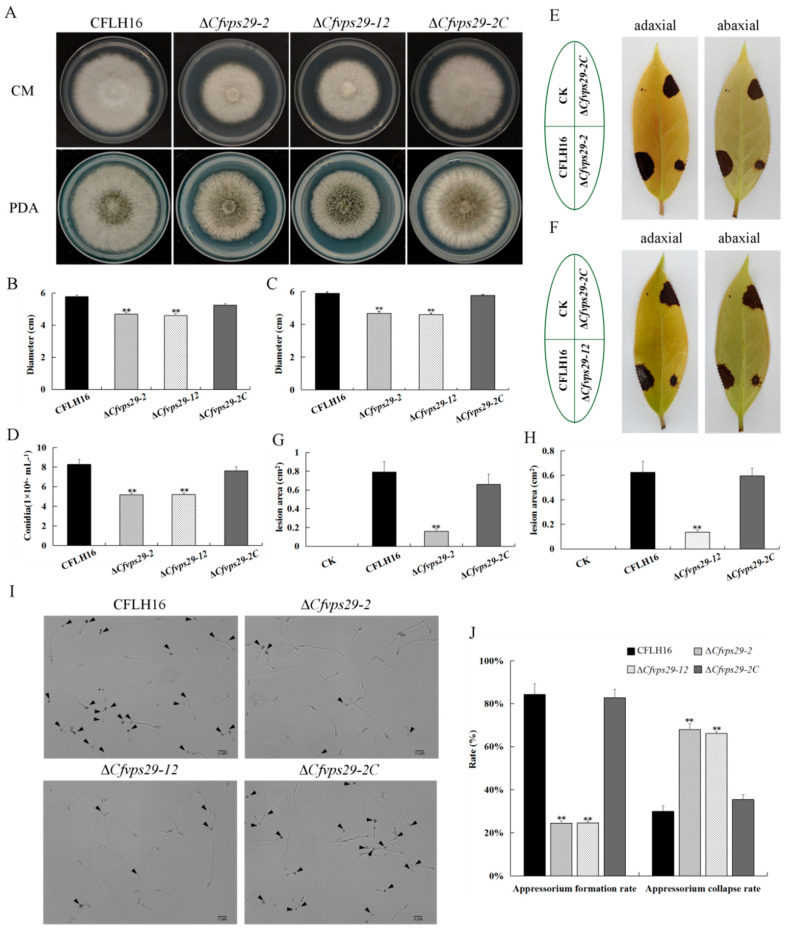
Phenotypic assays of the *CfVPS29* deletion mutants. (**A**) The wild-type (CFLH16), Δ*Cfvps29*-2, Δ*Cfvps29*-12, and the complemented strain Δ*Cfvps29-2C* were inoculated on CM and PDA. (**B**) Statistical analysis of the colony diameter variations on CM. (**C**) Statistical analysis of the colony diameter variations on PDA. (**D**) Conidia were measured and statistically analyzed by Duncan analysis. (**E**,**F**) Pathogenicity test strain CFLH16, ∆*Cfvps29*, and Δ*Cfvps29-2C* on wounded oil-tea leaves, CK indicates the negative control. (**G**,**H**) Statistical analysis of the lesion sizes on wounded leaves. (**I**) Appressoria formation in the wild-type (CFLH16), Δ*Cfvps29*-2, Δ*Cfvps29*-12, and the complemented strain Δ*Cfvps29-2C* (black arrow: appressorium). (**J**) Statistical analysis of the formation rate and collapse rate of the appressorium. All experiments were carried out at least three times, and each treatment had three replicates. Error bars represent the standard deviation and asterisks represent significance at *p* < 0.01 (**).

To determine whether the *CfVPS29* gene participates in the regulation of the vegetative growth of *C. fructicola*, the colony diameters of the wild-type, mutant, and complementary strains were measured and analyzed statistically after culture on CM and PDA plates. The data showed that the colony diameters of Δ*Cfvps29*-2 and Δ*Cfvps29*-12 were significantly smaller than those of the wild-type and complementary strains (average diameters on CM were: CFLH16: 5.77 cm, ∆*Cfvps29*-2: 4.68 cm, and ∆*Cfvps29*-12: 4.60 cm, ∆*Cfvps29*-2C: 5.25 cm; average diameters on PDA were: CFLH16: 5.90 cm, ∆*Cfvps29-2*: 4.67 cm, ∆*Cfvps29*-12: 4.60 cm, and ∆*Cfvps29*-2C: 5.77 cm) (*p* < 0.01, Figure 1A–C). Thus, it could be inferred that the *CfVPS29* gene is involved in regulating the vegetative growth of *C. fructicola*.

Next, the conidiation of the mutant strain was analyzed, revealing a significant reduction of about 40% in the sporulation ability compared to wild-type and complementary strains (Figure 1D). As a plant-pathogenic fungus, we concentrated on the role of *CfVPS29* in the pathogenicity of *C. fructicola*. We conducted a pathogenicity assay on wounded tea-oil tree leaves. The lesion area of the ∆*Cfvps29*-2 mutant was only 0.16 cm^2^, as opposed to 0.79 cm^2^ for wild-type and 0.66 cm^2^ for the complementary strain. The lesion area of the ∆*Cfvps29*-12 mutant was only 0.14 cm^2^, as opposed to 0.63 cm^2^ for the wild-type and 0.59 cm^2^ for the complementary strain (Figure 1E,F). Our results showed that the lesion areas caused by Δ*Cfvps29*-2 and Δ*Cfvps29*-12 were significantly smaller than those of the wild-type and complementary strains, respectively (Figure 1G,H, *p* < 0.01). The results showed that the *CfVPS29* gene participates in the pathogenesis of *C. fructicola*.

Based on the decrease in the pathogenicity of Δ*Cfvps29*, we wondered whether the mutant could not produce any functional appressorium. Further investigation showed that the rate of appressorium formation of the mutant strains Δ*Cfvps29*-2 and Δ*Cfvps29*-12 were only about 24% and 25% (Figure 1I), which was significantly lower than that of the wild-type and complementary strains (Figure 1J, *p* < 0.01).

Since the establishment and maintenance of high internal turgor pressure is necessary for appressorium-mediated host penetration, we examined the turgor pressure in Δ*Cfvps29* using the incipient cytorrhysis assays. In 2 M glycerol, 67% of Δ*Cfvps29* appressoria collapsed at 10 min, compared to 31% and 33% of the appressoria in the wild-type and complementation strains, respectively (Figure 1J, *p* < 0.01). These appressorial collapse assays revealed that the Δ*Cfvps29* appressoria generate significantly lower turgor compared to the wild-type and complementation strains. This result indicates that the *CfVPS29* gene is required for the functional appressorium formation of *C. fructicola.*

### 3.4. The CfVPS29 Gene Is Involved in the Maintenance of Cell Wall Integrity

**Figure 2:**
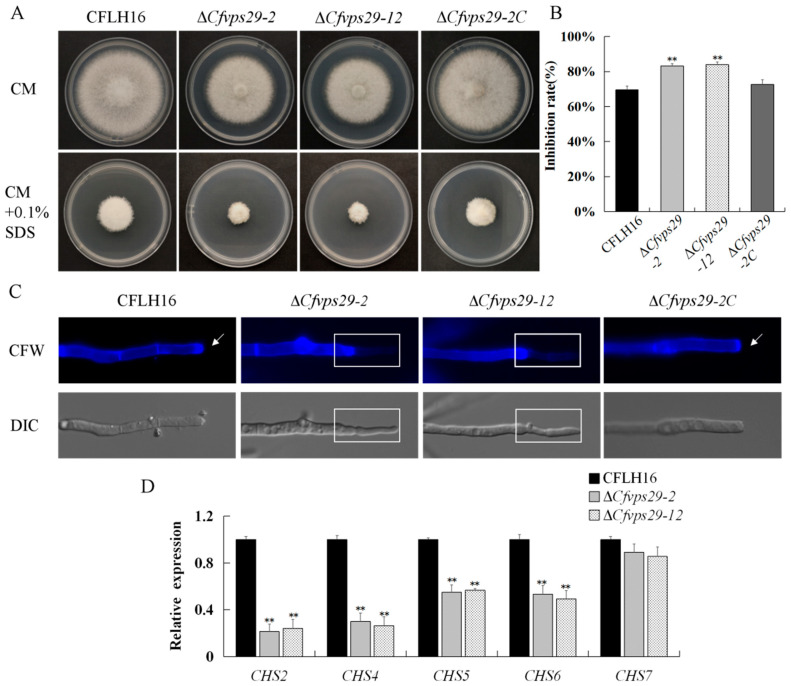
CfVps29 plays roles in cell wall integrity. (**A**) The wild-type (CFLH16), Δ*Cfvps29*-2, Δ*Cfvps29*-12, and the complemented strain Δ*Cfvps29-2C* were inoculated on complete medium (CM) containing 0.1% SDS. (**B**) Statistical analysis of the inhibition rates of the strains under cell wall stress, and asterisks indicating significant differences (*p <* 0.01). (**C**) The mycelia of the strains were stained with 10 mg/mL of CFW for 10 min; arrows indicate the stained hyphal tips; white boxs indicate no chitin distribution on the mycelium tip. (**D**) Reduced expression was found in four out of five *CHS* genes that encode chitin synthases in the Δ*Cfvps29* mutants. Error bars represent the standard deviation, and “**” represent significant differences among the stains tested. All of the reductions are significant (*p* = 0.01 or *p* = 0.05) according to Duncan’s multiple-range test. All experiments were carried out at least three times, and each treatment had three replicates, which showed the same results. DIC, differential interference contrast image.

The growth and development of *C. fructicola* were influenced by various environmental stresses in natural conditions. In this study, we tested the sensitivity of the strains under cell wall stress conditions. The inhibition rates of the mutant on sodium dodecyl sulfate (SDS) plates were significantly higher than those of the wild-type and complemented strains (Figure 2A,B). When treated with 0.1% SDS, ∆*Cfvps29*-2 and ∆*Cfvps29*-12 mutants showed inhibition rates of 83% and 84%, in contrast to those of 70% and 73% in the wild-type and complementary strains, respectively. These results indicate that the *CfVPS29* gene participates in the response of *C. fructicola* to cell wall stress.

The distribution of chitin in the cell walls of the ∆*Cfvps29* mutants was analyzed by Calcofluor white (CFW) staining. The results showed that the distribution of chitin on the mycelium tip was observed in the WT and complemented strains, but not in the ∆*Cfvps29*-2 and ∆*Cfvps29*-12 mutants (Figure 2C). Further analysis of the genes for chitin synthesis in the ∆*Cfvps29* mutants by RT-qPCR showed that the expression of the *CHS2*, *CHS4*, *CHS5*, and *CHS6* genes were significantly down-regulated (Figure 2D, *p* < 0.01), suggesting that the *CfVPS29* gene participates in the regulation of the chitin synthase gene expression in *C. fructicola*. In summary, these results revealed that *CfVPS29* is involved in the maintenance of cell wall integrity.

## 4. Discussion

The retromer complex is conserved across yeasts to animals and plants, and plays multiple roles in the physiological process [7]. *C. fructicola* is the dominant pathogen causing anthracnose in *Ca. oleifera* [1]. However, the function of Vps29 in *C. fructicola* is unclear. Here, we investigated the biological function of CfVps29 in *C. fructicola,* which orchestrates growth, development, and pathogenicity.

Appressorium is the key fungal structure infecting plant tissues. The appressorium penetrates the cuticle and cell wall of plants. In this study, we provided two direct reasons for the significantly reduced pathogenicity of the Δ*Cfvps29* mutant. Firstly, the appressorium formation rate of the Δ*Cfvps29* mutant was significantly reduced. In previous studies on *C. fructicola*, we found that the *CfSNF1*, *CfGCN5*, *CfVPS39*, and *CfVAM7* genes were involved in the regulation of appressorium development, and the mutants completely lost their pathogenicity [6,8,9,10]. CfVps29 may therefore be important for pathogenicity through its regulatory role in appressorium formation. Secondly, the Δ*Cfvps29* mutant showed significantly decreased appressorium turgor, which is essential for the appressorium-mediated host penetration [11]. The lower appressorium turgor of the Δ*Cfvps29* mutant may cause its pathogenicity defect. In the rice blast fungus *M. oryzae*, the *MoVPS29* gene is also required for conidiation and appressorial turgor generation [4]. The data suggest that the function of the retromer complex is fundamental for the appressorium formation and the internal turgor pressure in *C. fructicola*.

Chitin and glucan are two major components of the fungal cell wall. In plant pathogenic fungi, the development of the invasion structure, the growth, and morphology of mycelia depend on the regular synthesis and distribution of chitin in the cell wall [10,11,12]. In the rice blast fungus *M. oryzae*, different chitin synthase genes are required for various developmental and plant infection processes [13]. In *Aspergillus nidulans* classes V and VI, the chitin synthase csmA and csmB single deletion mutants showed no significant changes in the growth rate, but the csmA csmB double mutant was not viable [14], suggesting an essential role of these *CHS* genes in hyphal tip growth [15,16]. In our study, the ∆*Cfvps29* mutants showed higher sensitivity to the cell wall inhibitors. We further found that the absence of the accumulation of chitin at the hyphal tips in the ∆*Cfvps29* mutant strains, and the decreased genetic expression of chitin synthase, suggest that the retromer complex is necessary for correct the gene expression of chitin synthase and therefore for cell wall integrity. Moreover, the critical role of CfVps29 in the cell wall integrity suggests the function of the retromer complex in pathogenicity.

In conclusion, a *S. cerevisiae ScVPS29* homologous gene, *CfVPS29*, was identified and disrupted from *C. fructicola*. It was found that the *CfVPS29* gene plays a vital role in mycelial growth, sporulation, appressorium formation, the cell wall stress response, and the pathogenicity of *C. fructicola*. These results are useful in elucidating the pathogenic molecular mechanism of *C. fructicola* and providing potential targets for the development of new fungicides. To our knowledge, this study was novel in characterizing the function of CfVps29 in the fungal pathogens of cultivated woody species.

## Figures and Tables

**Table 1:** Primers used in this study.

Primer	Sequence (5′–3′)	Purpose
*CfVPS29*-1F	GGACAGAAGATTACACTGAG	amplify *CfVPS29* 5′ flank sequence
*CfVPS29*-2R	TTGACCTCCACTAGCTCCAGCCAAGCCGGTCGTTAGGGGTGTGTATA	amplify *CfVPS29* 5′ flank sequence
*CfVPS29*-3F	CAAAGGAATAGAGTAGATGCCGACCGGCCGTCATCATGCAAGACGA	amplify *CfVPS29* 3′ flank sequence
*CfVPS29*-4R	ACTGTCACGATCAAGCGCGA	amplify *CfVPS29* 3′ flank sequence
*CfVPS29*-5F	CATGTGTGCCTATGGCGTCA	validation of *CfVPS29* gene deletion
H855R	GCTGATCTGACCAGTTGC	validation of *CfVPS29* gene deletion
*CfVPS29*-7F	GCGCTCTTGATATCCCCCAA	validation of *CfVPS29* gene deletion
*CfVPS29*-8R	CACGGGCTTCGTGTAAGTCA	validation of *CfVPS29* gene deletion
*CfVPS29*-9F	ACTCACTATAGGGCGAATTGGGTACTCAAATTGGTTGATAACACGGACCTGTAGTG	amplify complemented sequence
*CfVPS29*-10R	CACCACCCCGGTGAACAGCTCCTCGCCCTTGCTCACTGATGTTGCAGACGGCTCCA	amplify complemented sequence
Hyg F	GGCTTGGCTGGAGCTAGTGGAGGTCAA	amplify HPH sequence
Hyg R	CGGTCGGCATCTACTCTATTCCTTTG	amplify HPH sequence
GFP-R	GACACGCTGAACTTGTGGCCGTT	validation of complemented sequence
*CHS2-F*	TCCGCCCCTCTGATTCCTAA	RT-qPCR
*CHS2-R*	ACATGAAGGAAGCCGCGTAA	RT-qPCR
*CHS4-F*	GAACATCGAGATGGCGCAAC	RT-qPCR
*CHS4-R*	CTCGCCGGACTCAGGTATTC	RT-qPCR
*CHS5-F*	CCCACAAGATGACGGACCTC	RT-qPCR
*CHS5-R*	GCGTCGAGGTAGAACTTGGT	RT-qPCR
*CHS6-F*	CAGTCTTGCCGCCTACATCA	RT-qPCR
*CHS6-R*	GTCGGCGTAGGAGTAAGCTC	RT-qPCR
*CHS7-F*	GCAAATTCACCGCTGTTGGT	RT-qPCR
*CHS7-R*	CAGCATACACGGAGAAGCCA	RT-qPCR
*ACTIN-F*	CCCCATCTACGAGGGTTTCG	RT-qPCR
*ACTIN-R*	CGTCAGGAAGCTCGTAGGAC	RT-qPCR

## Data Availability

All data generated or analyzed during this study are included in this published article and its supplementary materials.

## Supplementary Materials

The following supporting information can be downloaded at: https://www.mdpi.com/article/10.3390/jof8080835/s1, Figure S1: Phylogenetic analysis and domain prediction of CfVps29. (A) The number on the branch is the % bootstrap support value; the scale bar indicates the branch length. The GenBank accession numbers are shown as follows: *C. truncatum* (XP 036584255.1); *C. karsti* (XP 038739603.1); *Pyricularia oryzae* (XP 003709334.1); *Fusarium austroafricanum* (KAF4450725.1); *S. cerevisiae* (NP011876.1); *C. fructicola* (XP_031883282.1). (B) The protein motif arrangements of CfVps29 and its homologues. Different colors represent different motifs. Straight lines represent total protein length. (C) The protein domain arrangements of CfVps29 and its homologues are visualized in the schematic at the right. Straight lines represent total protein length. (D) Six motif consensus sequences. Figure S2: Generation of the gene deletion mutant of *CfVPS29*. (A): Schematic of the deletion strategy. (B): Electrophoretic gel for verification. negative control: ddH_2_O, positive control: wild type strain.
