# Supplementary material for: The Retromer Subunit CfVps29 Is Involved in the Growth, Development, and Pathogenicity of Colletotrichum fructicola
Source: J Fungi (Basel). 2022 Aug 10;8(8):835. doi: 10.3390/jof8080835 (PMC9409673; doi:10.3390/jof8080835)
Supplement: Supplementary file 1 [file jof-08-00835-s001.zip › jof-1820262-supplementary.pdf]

Supplementary Information

The retromer subunit CfVps29 is involved in the growth, development and pathogenicity of *Colletotrichum fructicola*

Li Sizheng et al.

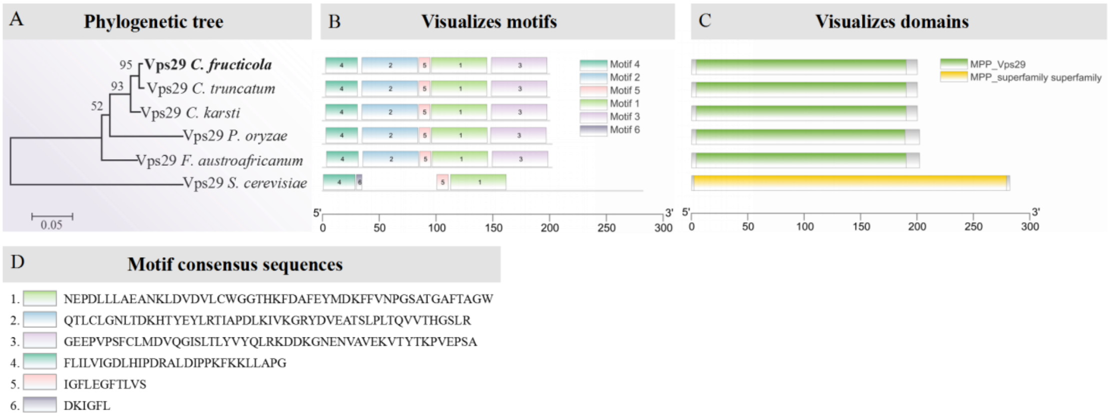

Figure S1. Phylogenetic analysis and domain prediction of CfVps29. (A) The number on the branch is the % bootstrap support value; the scale bar indicates the branch length. The GenBank accession numbers are shown as follows:*C. truncatum*(XP 036584255.1); *C. karsti* (XP 038739603.1); *Pyricularia oryzae* (XP 003709334.1); *Fusarium austroafricanum* (KAF4450725.1); *S. cerevisiae* (NP 011876.1); *C. fructicola* (XP_031883282.1). (B) The protein motifs arrangements of CfVps29 and its homologues. Different colors represent different motifs. Straight lines represent total protein length. (C) The protein domain arrangements of CfVps29 and its homologues are visualized in the schematic at the right. Straight lines represent total protein length. (D) Six motif consensus sequences.

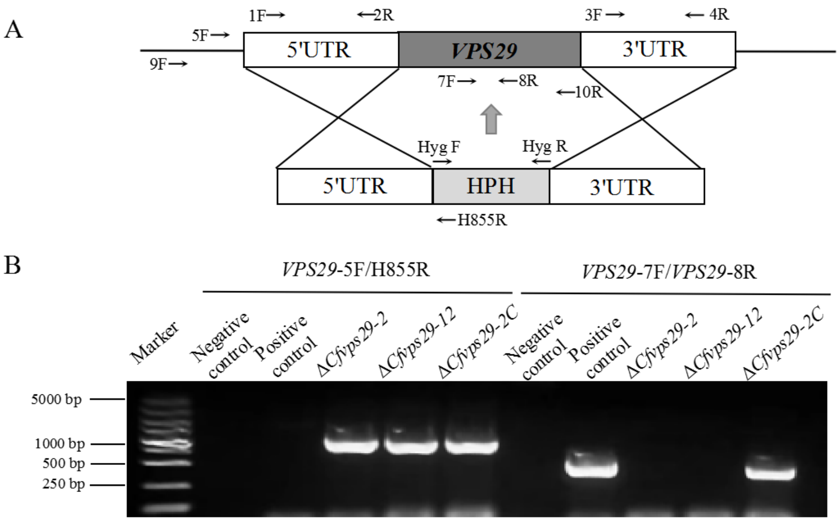

Figure S2. Generation of the *CfVPS29* gene deletion mutant. (A): Schematic of the deletion strategy; (B): Electrophoretic gel for verification. negative control: ddH_2O, positive control: wild type strain.
